# Impacts of ciliary neurotrophic factor on the retinal transcriptome in a mouse model of photoreceptor degeneration

**DOI:** 10.1038/s41598-020-63519-1

**Published:** 2020-04-20

**Authors:** Yanjie Wang, Kun-Do Rhee, Matteo Pellegrini, Xian-Jie Yang

**Affiliations:** 1 Department of Ophthalmology and Stein Eye Institute, University of California, Los Angeles, CA USA; 2 Department of Molecular, Cell and Developmental Biology, University of California, Los Angeles, CA USA

**Keywords:** Eye diseases, Molecular biology, Neuroscience, Molecular medicine

## Abstract

Ciliary neurotrophic factor (CNTF) has been tested in clinical trials for human retinal degeneration due to its potent neuroprotective effects in various animal models. To decipher CNTF-triggered molecular events in the degenerating retina, we performed high-throughput RNA sequencing analyses using the *Rds/Prph2 (P216L)* transgenic mouse as a preclinical model for retinitis pigmentosa. In the absence of CNTF treatment, transcriptome alterations were detected at the onset of rod degeneration compared with wild type mice, including reduction of key photoreceptor transcription factors *Crx*, *Nrl*, and rod phototransduction genes. Short-term CNTF treatments caused further declines of photoreceptor transcription factors accompanied by marked decreases of both rod- and cone-specific gene expression. In addition, CNTF triggered acute elevation of transcripts in the innate immune system and growth factor signaling. These immune responses were sustained after long-term CNTF exposures that also affected neuronal transmission and metabolism. Comparisons of transcriptomes also uncovered common pathways shared with other retinal degeneration models. Cross referencing bulk RNA-seq with single-cell RNA-seq data revealed the CNTF responsive cell types, including Müller glia, rod and cone photoreceptors, and bipolar cells. Together, these results demonstrate the influence of exogenous CNTF on the retinal transcriptome landscape and illuminate likely CNTF impacts in degenerating human retinas.

## Introduction

Retinal degeneration (RD) is known as an irreversible, progressive neurologic disorder caused by genetic mutations and/or environmental or pathological damage to the retina. One therapeutic strategy to attenuate vision loss in disease conditions is treating the retina with neuroprotective agents that promote neuronal viability. Ciliary neurotrophic factor (CNTF) has been shown to be a potent neuroprotective agent for various types of retinal degenerations, including those caused by gene mutations, light-induced damage, and physical injury^1^. Since CNTF enhances the survival of both photoreceptors and retinal ganglion cells, the two major cell classes damaged in retinal degenerative diseases, a CNTF-based therapy has been developed and tested in several clinical trials. To date, outcomes of the trials for retinitis pigmentosa, geographic atrophy, and achromatopsia have not demonstrated sufficient therapeutic benefits for human patients^2–5^. However, CNTF therapy for macular telangiectasia type 2 has shown efficacy^6,7^, and a clinical trial for the second largest blinding disease glaucoma^8^ is ongoing (https://clinicaltrials.gov/ct2/show/NCT01408472).

To shed light on the molecular and cellular mechanisms of CNTF-mediated neuroprotection, we have delivered the same secreted recombinant CNTF used in human trials to a mouse model expressing the *Rds/peripherin2* (*Prph2*) transgene with the *P216L* mutation^9^. Pheripherin2 is a structural protein of the photoreceptor outer segment disc, and over 80 *PRPH2* pathological mutations have been linked to RD, with clinical presentations ranging from retinitis pigmentosa to various forms of macular degeneration^10^. Transgenic mice expressing the *P216L* mutant peripherin2 protein exhibit progressive retinal degeneration with rod cell death preceding cone photoreceptor loss, mimicking the dominant disease feature observed in retinitis pigmentosa patients carrying this missense mutation^9^. In the mammalian retina, CNTF activates both the Jak-STAT and MEK-ERK signaling pathways^11^ through binding to a tripartite receptor complex consisting of gp130, LIFRβ, and CNTFRα, with *Cntfr* uniquely expressed in the nervous system^12^. By performing cell type-specific *gp130/Il6st* gene deletions in the *Rds/Prph2(P216L)* mutant retina, we have previously identified the initial target cells for exogenous CNTF as Müller glia^13^. We have also shown that the gp130 receptor is subsequently required directly by rod cells for CNTF-dependent survival^13^. Existing evidence supports that exogenous CNTF triggers amplification of a signaling loop between Müller glial cells and photoreceptors, which is required for CNTF-mediated neuroprotection in degenerating retinas^13^.

Despite the promise of CNTF as a broad-spectrum neuroprotective agent to attenuate retinal degeneration, we and others have observed that prolonged exposure to high levels of CNTF can be detrimental to visual function in animal models of RD^14–17^. To understand the effects of exogenous CNTF at the molecular level, we have applied high throughput RNA-sequencing (RNA-seq) technology to examine CNTF-induced alterations of the retinal transcriptome in this study. By comparing wild type and the *Rds/Prph2(P216L)* transgenic mouse, which serves as a preclinical model for retinitis pigmentosa, we captured transcriptome alterations due to the *Rds/Prph2* mutation at the onset and during the course of photoreceptor degeneration. Our RNA-seq analyses revealed dynamic gene expression patterns following short-term (3 and 24 hours) CNTF treatments and sustained modifications of the transcriptome landscape after long-term (10 days) CNTF exposure. Furthermore, we compared bulk RNA-seq results with an available single cell RNA-seq data to decipher retinal cell types affected by CNTF treatment. We also validated our RNA-seq data by comparison with other RD mouse models and available proteomic data. Together, results of these molecular analyses demonstrate the impacts of CNTF treatments on the retinal transcriptome in diseased conditions, thus providing valuable insight for CNTF clinical trials aimed at attenuating retinal degeneration in patients.

## Results

### Short-term CNTF treatment-induced transcriptome changes

**Figure 1:**
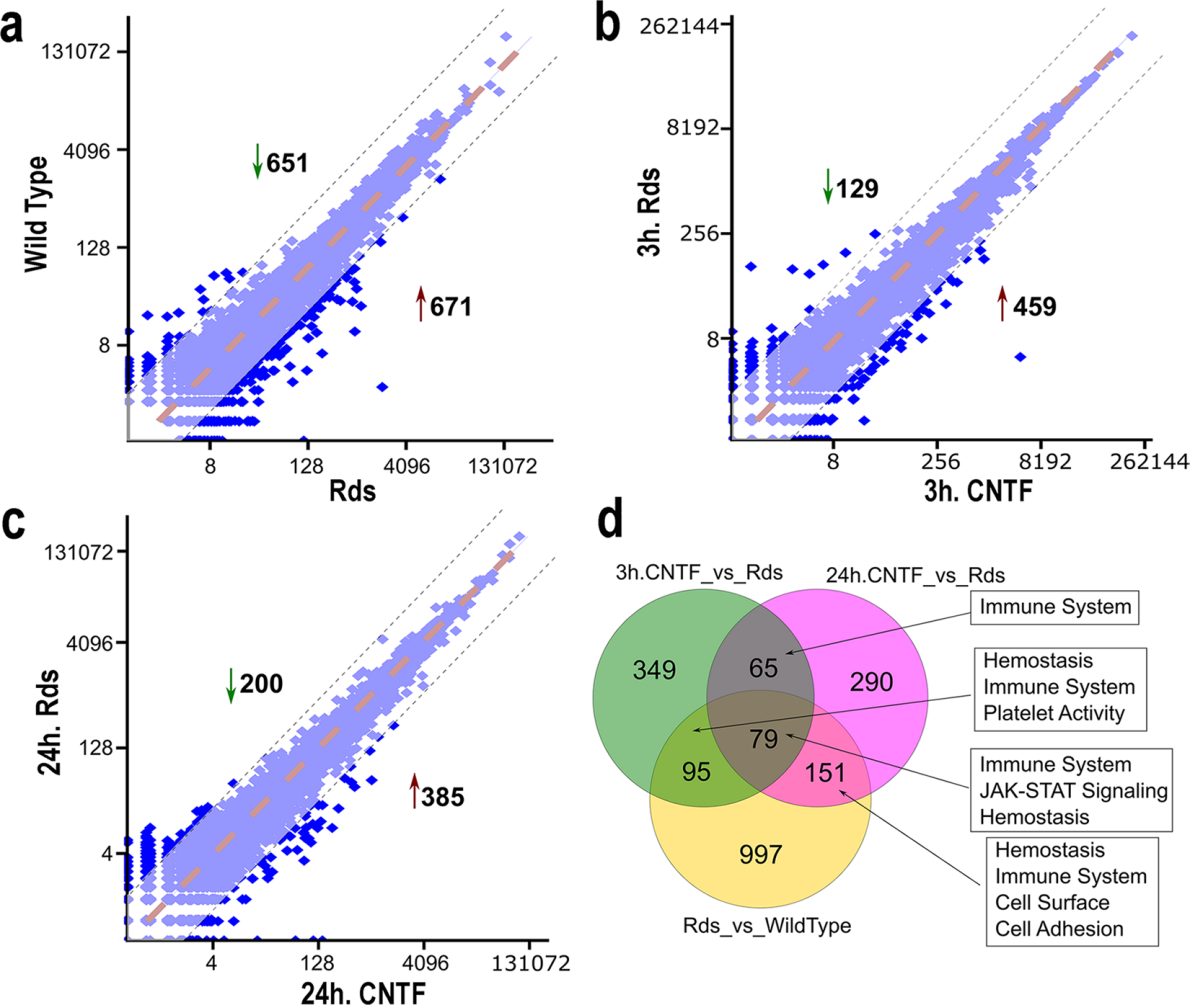
Assessment of differential expression after short-term CNTF treatments. Scatterplots show comparison of gene expression levels at P25 for (**a**) *Rds* mutant versus wild type, (**b**) 3-hour CNTF treatment versus PBS injections in the *Rds* mutant, (**c**) 24-hour CNTF treatment versus PBS injections in the *Rds* mutant. (**d**) Venn diagram shows the overlaps of up- and down-regulated genes and the main gene signatures among wild type, *Rds*, and CNTF-treated *Rds* samples. All data sets include genes *p* values < 0.01 in Fisher’s Exact Test.

To determine effects of the RD mutation-induced perturbation, we performed RNA-seq analyses and compared retinal-transcriptomes of wild type and *Rds/Prph2*(*P216L)*^9^ (hereafter referred to as *Rds*) mice at postnatal day 25 (P25) (Supplementary Table S1). To examine the acute impact of exogenous CNTF on retinal gene expression, we also analyzed *Rds* retinas after short-term CNTF treatments for 3 hours or 24 hours with parallel PBS-injected Rds retinas as controls (Supplementary Table S1). RNA-seq data analyses revealed differentially expressed genes between matched sample pairs (Fig. 1). At P25, when the onset of retinal degeneration had begun in the *Rds* retina, the mutant retina exhibited significant differences in mRNA expression from wild type littermates for 1322 genes, with 651 genes downregulated and 671 genes upregulated (Fig. 1a). After 3-hour treatment of the *Rds* mutant, the CNTF-induced response was readily detected in transcript levels of 588 genes compared to vehicle-injected mutant retinas as controls (Fig. 1b). After 24-hour CNTF treatment, 585 genes in total showed significantly altered expression compared to vehicle-injected *Rds* mutant controls (Fig. 1c). Furthermore, we observed overlapping genes among the significantly altered transcripts in the three sample pairs (Fig. 1d). Among altered transcripts between the *Rds* and wild type retinas, we found 325 genes also corresponded to short-term CNTF treatment-induced changes, suggesting that the *Rds* mutation and short-term CNTF treatments shared cellular processes and responses. Interestingly, the gene sets between the 3-hour and 24-hour CNTF treatments were not identical, with an overlapping subset of only 144 genes, reflecting the dynamic retinal transcriptome response to exogenous CNTF signals.

**Figure 2:**
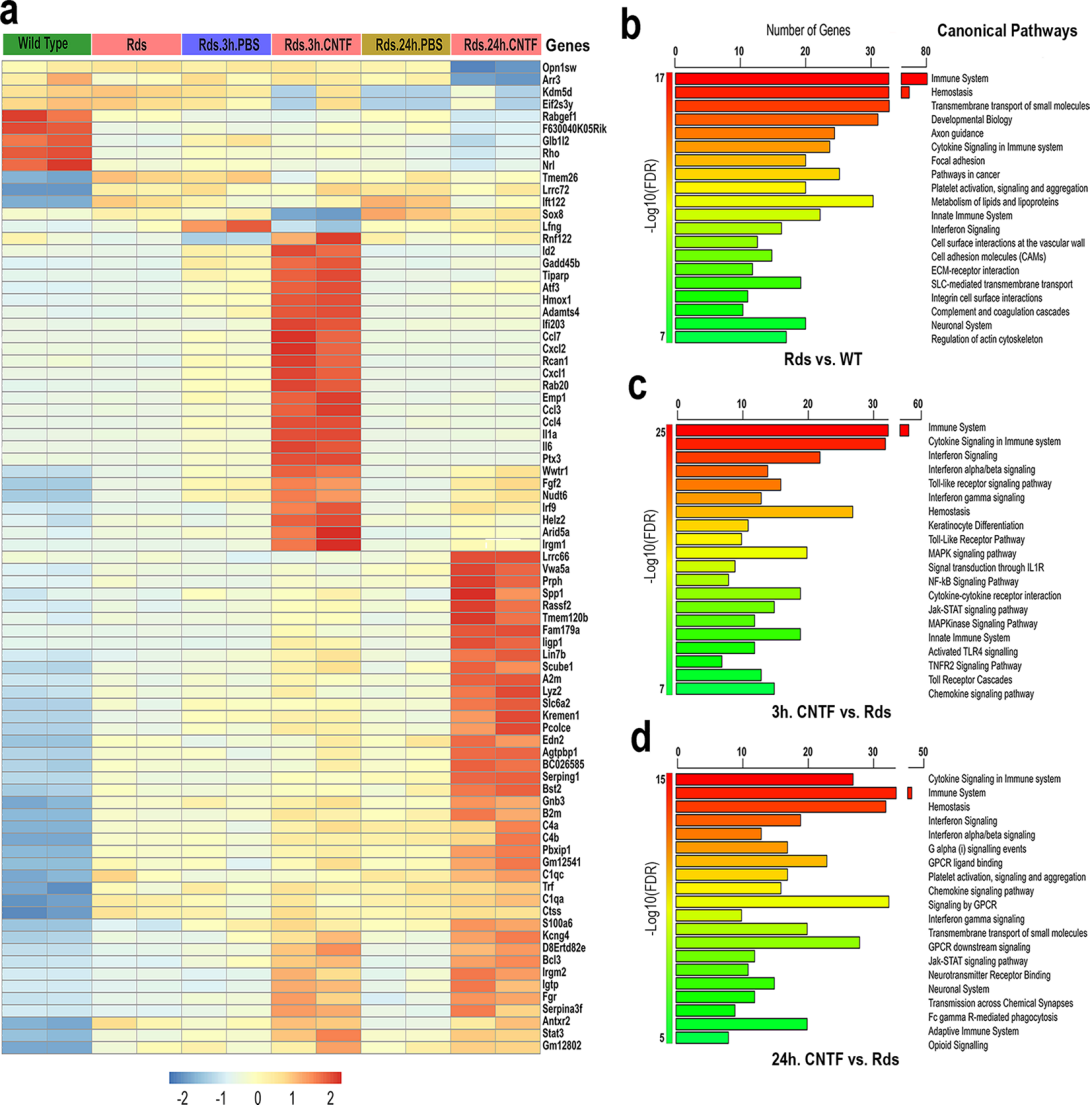
Top gene signatures and pathway enrichment following short-term CNTF treatments. (**a**) Heatmap of the top 30 differentially expressed genes (DEGs) according to DESeq tests is shown for duplicating samples. The colors are scaled by row, with red and blue indicating 2 standard deviations above or below the mean (white), respectively. The enriched pathways from the Molecular Signatures Database (MSigDB) are shown for (**b**) *Rds* mutant versus wild type, (**c**) 3-hour CNTF treatment versus PBS injections in the *Rds* mutant, (**d**) 24-hour CNTF treatment versus PBS injections in the *Rds* mutant. The top 20 pathways satisfying false discovery rate (FDR) < 0.01 are presented. The horizontal axis shows the number of genes represented in each pathway.

We next compared the top 30 gene signatures for the three matched sample sets of the short-term CNTF treatments (Fig. 2; Supplementary Table S2). At the onset of degeneration, the P25 *Rds* mutant already exhibited significant changes in gene expression compared to the wild type retina. As controls for both short-term treatments, the PBS-injected *Rds* retinas largely retained the transcriptome landscape of the non-injected *Rds* mutant (Fig. 2a). However, the 3-hour and 24-hour CNTF treatments both resulted in distinct transcriptome alterations (Fig. 2a). We observed several cytokines and growth factors with altered expression during an initial 3-hour CNTF-induced transcription wave, including *IL1a, IL6, Cxcl2, Ccl3, Ccl4, Ccl7*, and *Fgf2*. However, this early wave of induced transcripts was diminished within 24 hours and replaced by a different group of elevated transcripts, which included multiple components of the complement system, such as *C1qa, C4a* and *C4b*. The two short-term samples also shared common transcripts, including *Irgm2*, *Igtp, D8Ertd82e*, *Bcl3*, and *Serpina3f*, which were further elevated compared to their corresponding controls. Most of these common genes participate in immune and complement pathways^18–20^.

We used MsigDB, a component of Gene Set Enrichment Analysis (GSEA), to further investigate enrichment of the detected gene signatures during short-term CNTF perturbations (Fig. 2b–d). The analysis showed that genes related to the immune system, cell-matrix interactions, and developmental processes were already overrepresented in *Rds* versus wild type retinas (Fig. 2b). After short-term CNTF exposure, transcripts associated with immune pathways (e.g. cytokine and interferon signaling), neuronal processes (transmission across chemical synapses and neurotransmitter receptor binding, etc.), growth factor signaling, GPCR signaling, and small molecule transporter systems became the dominant gene signatures (Fig. 2c,d). We also explored the directionality (up or down regulation) of gene expression at the pathway level. The up-regulated gene signatures in short-term CNTF treated *Rds* retinas were mostly associated with immune-related extracellular growth factor signaling pathways, whereas the down-regulated genes were frequently involved in GPCR signaling and neuronal system processes (Supplementary Table S3).

### Rapid impact on cytokine signaling and photoreceptor transcription

**Figure 3:**
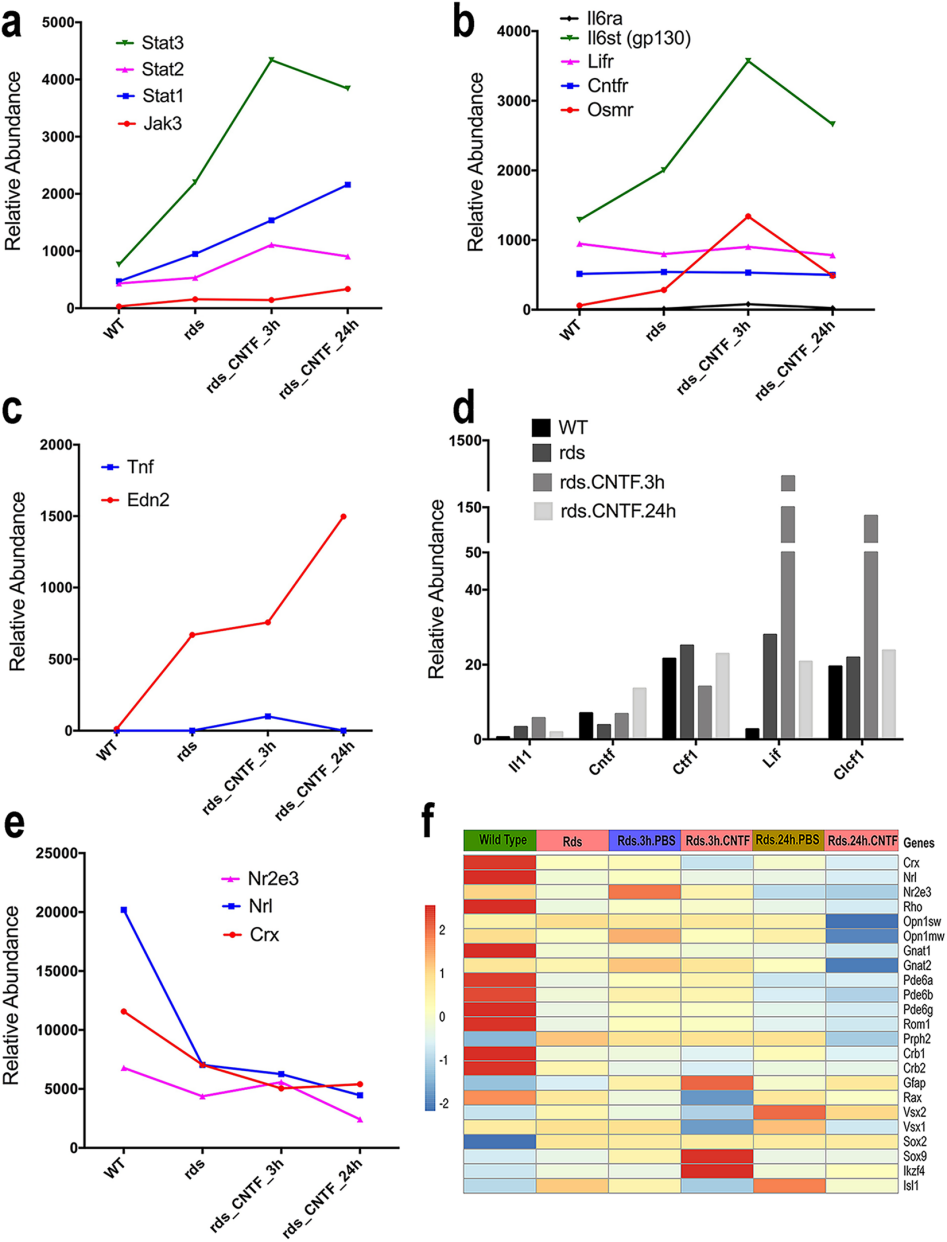
Effects of short-term CNTF treatments on expression of cytokine signaling and retinal genes. Relative retinal transcript levels in wild type, *Rds*, and *Rds* mutant after short-term CNTF treatments are shown for (**a**) Jak-STAT pathway genes, (**b**) cytokine receptor components, (**c**) TNF alpha and Endothelin 2, (**d**) CNTF subfamily of cytokines, (**e**) key photoreceptor transcription factors, (**f**) a heatmap for a selected subset of retinal genes, with the red and blue color scaled in row to indicate 2 standard deviations above or below the mean (white), respectively.

We next examined the expression of cytokine signal transduction components and photoreceptor-specific genes (Fig. 3). CNTF rapidly induced expression of the *Stat* family of genes, especially *Stat3* and *Stat1* (Fig. 3a). In addition, CNTF augmented cytokine receptor *Il6st/gp130* expression within 3 hours (Fig. 3b). Within a 24-hour period, CNTF transiently stimulated *Tnfa* expression, while substantially elevated expression of *Edn2*, a factor induced in a variety of retinal degeneration conditions^21^ (Fig. 3c). The RNA-seq data also confirmed that *Lif* was upregulated in the *Rds* mutant compared to the wild type retina (Fig. 3d). Furthermore, transient spikes of *Lif* and *Clcf1*, two members of the CNTF subfamily of cytokines, occurred after a 3-hour CNTF stimulation, but quickly declined to non-treated *Rds* mutant levels within 24 hours (Fig. 3d; Supplementary Table S2)^13^.

Among the key transcription factors involved in maintaining photoreceptor identities^22–26^, the *Rds* mutant retina showed reduced expression of *Crx* by 39% (p < 4.48E-05), *Nrl* by 65% (p < 8.26E-22), and *Nr2e3* by 35% (p < 0.01) compared to the wild type retina at P25 (Fig. 3e). A number of rod-specific genes also showed considerably lower levels of expression in the *Rds* mutant retinas compared to wild type retinas, including *Rho (*31%, p < 1.80E-31), *Gnat1* (44%, p < 2.38E-16), and *Pde6b* (47%, p < 5.07E-14) (Fig. 3f, Supplementary Table S4). As expected, expression levels for the cone-specific genes *Opn1sw, Opn1mw*, and *Gnat2* in the *Rds* mutant retinas at P25 did not deviate from wild type levels since cone degeneration is delayed in this retinitis pigmentosa model (Fig. 3f). Short-term CNTF treatments caused further reductions of *Crx, Nrl*, and *Nr2e3* in the *Rds* retina (Figs. 3e,f, Supplementary Table S4). Consistent with these observations, RNA-seq analyses detected further reduced expression of the rod-specific *Rho* (29%, p < 0.00023), *Gnat1* (24%, p < 0.0034), and *Pde6b* (23%, p < 0.0061) after a 24-hour exposure to CNTF (Fig. 3f, Supplementary Table S4). Moreover, 24-hour CNTF treatments resulted in rapid down-regulation of many cone-specific genes, including *Opn1sw* by 77% (p < 5.13E-45), *Opn1mw* by 44% (p < 1.68E-07), and *Gnat2* by 34% (p < 5.25E-0.5) (Fig. 3f, Supplementary Table S4). In contrast, *Gfap* and *Vsx2* mRNAs were increased in the *Rds* mutant as a consequence of CNTF stimulation (Fig. 3f, Supplementary Table S4). These results demonstrate that exogenous CNTF causes dynamic changes to the retinal transcriptome landscape within a short time window in the *Rds* degenerating retina and asserts a strong impact on photoreceptor gene transcripts.

### Influence on transcriptome by long-term CNTF treatment

**Figure 4:**
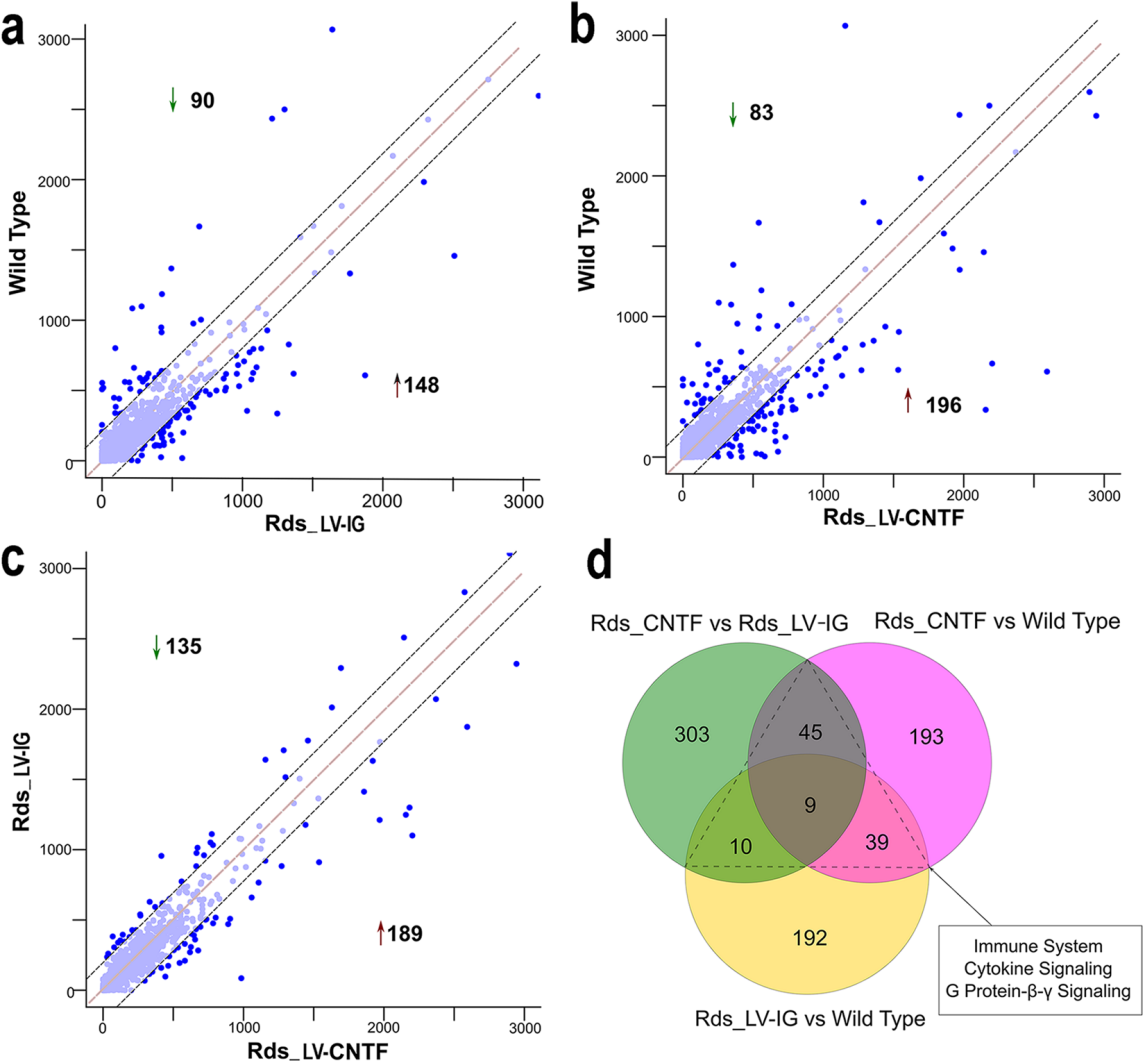
Assessment of differential expression after the long-term CNTF treatment. Scatterplots show comparisons of retinal gene expression after control or CNTF virus transduction from P25 to P35 for (**a**) wild type versus *Rds* mutant transduced with LV-IG, (**b**) wild type versus *Rds* mutant transduced with LV-CNTF, (**c**) LV-CNTF versus LV-IG treated *Rds* retinas. (**d**) Venn diagram shows the overlaps of up- and down-regulated genes among wild type and virus-transduced *Rds* retinas. All data sets include genes with *p* values < 0.01 in Fisher’s Exact Test.

To study the long-term effects of CNTF treatment on retinal transcriptomes, we expressed the recombinant human CNTF *in vivo* using a lentiviral vector LV-CNTF, which expresses the same secreted form of recombinant CNTF used in clinical trials^13^. Since subretinal delivery of LV-CNTF at P25 results in substantial rescue of photoreceptors in the *Rds* mutant by P35^13^, we performed RNA-seq profiling of wild type, and *Rds* mutant retinas transduced with either LV-CNTF or a GFP-expressing control virus LV-IG from P25 to P35 (Supplementary Table S1). Retinal transcriptome analyses for differential expression detected 238 genes between wild type and *Rds* mutant retinas infected with LV-IG, 279 genes between wild type and *Rds* mutant retinas infected with LV-CNTF, and 324 genes between LV-IG and LV-CNTF virus infected *Rds* retinas (Fig. 4; Supplementary Table S5).

**Figure 5:**
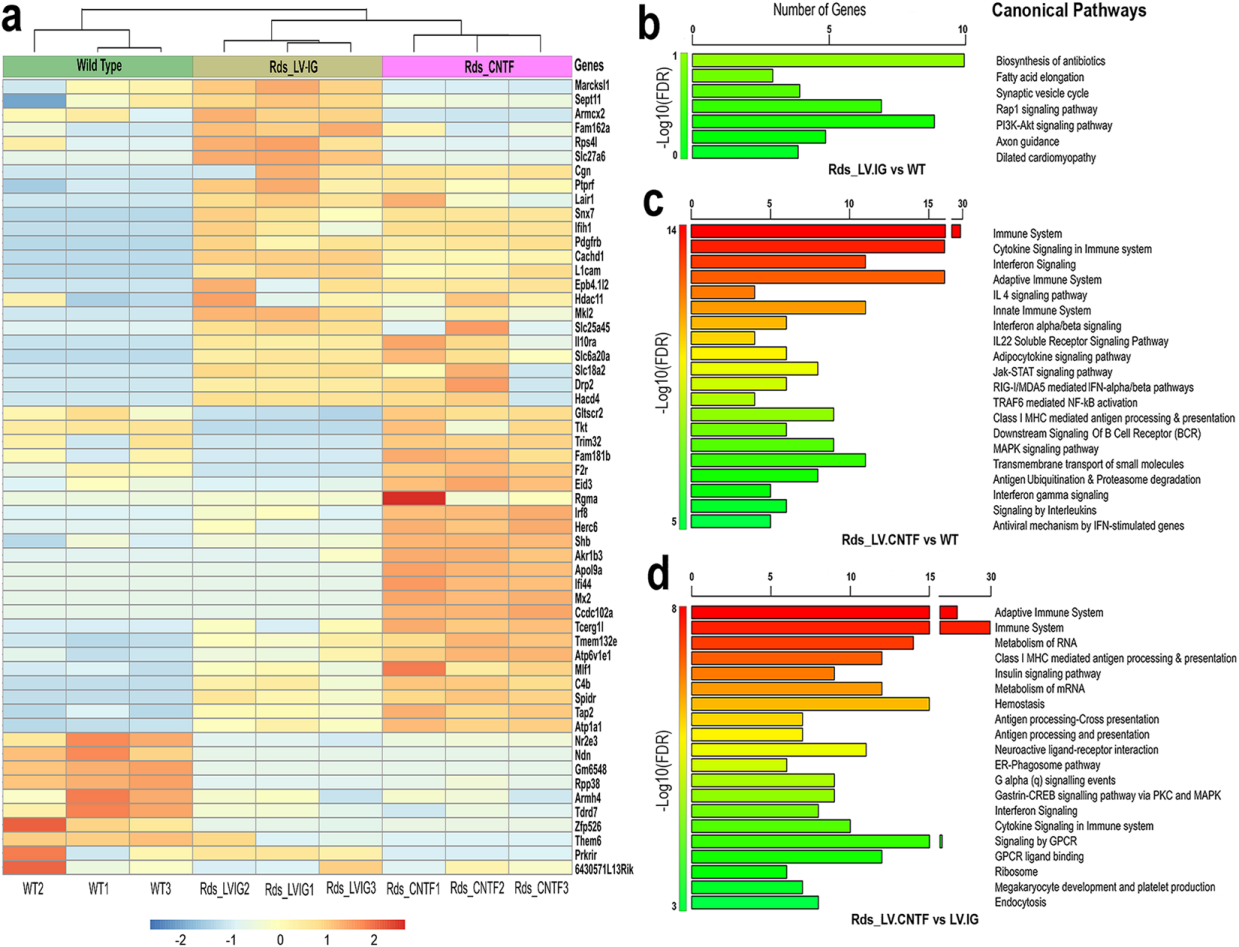
Top gene signatures and enriched pathways after the long-term CNTF treatment. (**a**) A heatmap shows the top 30 DEGs according to DESeq in triplicates for wild type and Rds retinas transduced with the control virus LV-IG or LV-CNTF. The colors are scaled by row, with red and blue indicating 2 standard deviations above or below the mean (white), respectively. The enriched pathways from the Molecular Signatures Database (MSigDB) are shown for (**b**) wild type versus *Rds* transduced with LV-IG, (**c**) wild type versus *Rds* transduced with LV-CNTF, (**d**) LV-CNTF versus LV-IG transduced *Rds* retinas. The top pathways (up to 20) satisfying false discovery rate (FDR) < 0.01 are presented. The horizontal axis shows the number of genes represented in each pathway.

Analyses of the top gene signatures for each pair-wise comparison revealed shared gene expression trends between LV-CNTF and control virus transduced *Rds* retinas, as well as distinct gene signatures associated with long-term CNTF treatment (Fig. 5a). Pathway enrichment analyses showed that when compared to wild type retinas, the P35 *Rds* mutant transduced with LV-IG displayed alterations in gene signatures related to Rap1 and PI3k-Akt signaling, synaptic vesicle cycling, axonal guidance, and fatty acid elongation (Fig. 5b). Predominant differential gene signatures between P35 *Rds* retinas treated with LV-CNTF and wild type retinas were related to the adaptive and innate immune systems (Fig. 5c). For comparison between LV-CNTF and LV-IG transduced *Rds* retinas, long -term CNTF treatment retained the top gene signatures related to the immune system as observed with the short-term CNTF exposure (Fig. 5d). In addition, long-term CNTF treatment resulted in transcript enrichment for genes associated with cellular homeostasis, RNA metabolism, insulin signaling, and transcription/translation processes, whereas genes involved in neuronal processes, such as chemical synapse transmission and neuroactive ligand receptor interactions, were down-regulated (Fig. 5d; Supplementary Table S6).

### Commonality of retinal transcriptome changes among degeneration models

**Figure 6:**
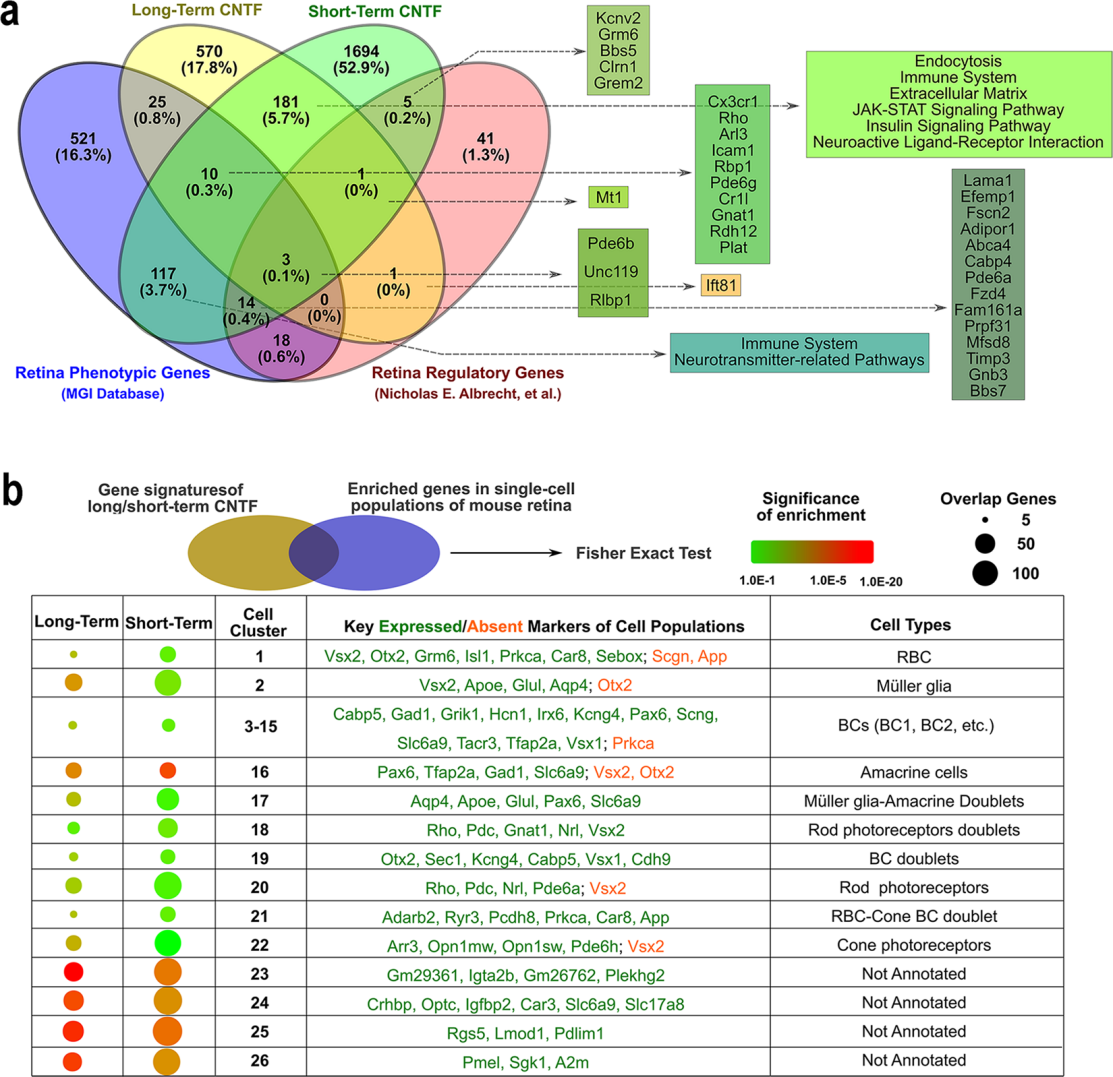
Enriched gene signatures of CNTF-treated retina and retinal cell types affected by CNTF. (**a**) Venn diagram demonstrates expression overlaps of short-term (3-hour and 24-hour) and long-term CNTF-treated *Rds* retinas with previously identified retinal phenotypic genes and retinal regulatory genes. Predominantly enriched pathways in overlapping signature are indicated. (**b**) CNTF-induced gene signatures of cell populations were compared with a retrieved single cell RNA-sequencing database to identify retinal cell types affected by CNTF. Fisher’s Exact tests (all *p* values < 0.01) were performed to examine the enrichment of gene signatures detected in the study. RBC, rod bipolar cell; BC, bipolar cell.

In order to search for common transcriptome features for retinal degeneration, we explored existing databases relevant to our RNA-seq analyses (Supplementary Table S7). First, we retrieved retina phenotypic genes from the MGI database and found expression overlaps with both our short- and long-term CNTF treatment-induced gene signatures (Fig. 6a). In addition, we detected signatures that overlapped between CNTF-dependent genes and retina regulatory genes^27^ (Fig. 6a). Second, we compared the transcriptomes of the *Rds* mouse model used in this study with RNA-seq data from two other retinal degeneration models, the *BC027072* knockout mouse^28^ and the *Rd10* mouse^29^ (Supplementary Table S8). The top gene signatures of the *Rds* mutant at P25 and P35 showed significant overlap with these two other disease models^28,29^ (Supplementary Fig. S1). For instance, 497 out of 1079 (46.1%) signature genes from the *Rd10* mouse overlapped with *Rds* mutant signature genes that deviated from wild type (Supplementary Fig. S1). The common enrichments included genes in immune pathways and neurotransmitter-related pathways, suggesting that *Rd10* and *Rds* mutants shared perturbations in similar biological processes during photoreceptor degeneration.

In order to gain insight into potential changes at the protein level in *Rds* mutant mice under the influence of CNTF, we analyzed and cross-referenced existing proteomic data from the *Rd10* model during degeneration^30^. Many gene transcripts significantly impacted by both short- or long-term CNTF treatments in the *Rds* retina were also detected in the *Rd10* retinal proteome (2600 proteins in the pool) at pre-, peak-, and post-degeneration time points, with 69 gene signatures matching *Rd10* protein signatures that deviated from wild type at any time point (Supplementary Fig. S2)^30^. In particular, we observed the same declining trend of photoreceptor genes and proteins, including Crx, Rho, Gnat1, Pde6b, Pde6g, beginning at the peak of degeneration, and becoming more severe at the post-degeneration stage. Conversely, a large set of gene transcripts induced in CNTF-treated *Rds* retinas matched the increased protein levels observed in the degenerated *Rd10* retina, including the chemokine signaling pathway genes STAT1, STAT3, ROCK2, GRK1, and GFAP.

### Impacts of CNTF treatment on different retinal cell types

We have shown previously that after one-hour exposure to exogenous CNTF, the Jak-STAT signaling pathway is predominantly activated in Müller glial cells in the mouse retina^13^. However, by 24 hours post CNTF exposure, the signaling events are no longer restricted to Müller glia, but instead have propagated throughout the entire retina. We thus explored whether CNTF-induced transcriptome alterations affected different retinal cell types. We cross-checked our bulk RNA-seq data with the single cell RNA-seq data across ~28,000 mouse retinal cells^31^. This analysis revealed that several different cell types are affected by exposure to exogenous CNTF (Fig. 6b), including Müller glia, rod and cone photoreceptors, bipolar cells, and amacrine cells, thus suggesting that diverse cell types and molecular mechanisms contribute to the effects of CNTF on the retinal transcriptome.

## Discussion

Phototransduction and the visual cycle are important functions in sensing and converting light signal into biochemical and electrical signals in the retina. Perturbations or mutations of the molecules responsible for these visual processes cause several types of RD. There is great genetic and allelic heterogeneity associated with the various retinal dystrophies^32^. Animal models, especially mice carrying gene mutations, have been used as powerful tools to investigate the etiology of human RD^33–35^. RNA-Seq is an extremely high-throughput approach compared with previously available analytic methods, providing the foundation for novel genetic discovery and identification of potential therapeutic targets in the treatment of RD^36^. To understand the molecular mechanism underlying CNTF-mediated neuroprotection, we performed RNA-seq analyses using a well-established preclinical model of retinitis pigmentosa. Our study generated RNA-seq transcriptomes from mouse retinas under different physiological conditions, including wild type, the *Rds/Prph2(P216L)* mutant, and the *Rds* mutant exposed to either short- or long-term CNTF treatments. Our results revealed deviations between the *Rds* and the wild type transcriptomes and commonalities shared among different mouse RD models. In addition, our data captured the retinal transcriptome landscape remodeling that occurs in response to acute and prolonged CNTF treatments in the degenerating retinas.

Through RNA-seq analyses of short-term CNTF treatment, we captured a dynamic transcriptome landscape. Consistent with the progression of photoreceptor degeneration in retinitis pigmentosa, the RNA-seq data showed that at an early stage of degeneration in the *Rds* retina (P25), the expression levels of rod-specific genes *Crx, Nrl, Rho*, *Gnat1, Pde6a, Pde6e, and Pde6g* had already decreased, while the expression levels for cone-specific genes had not been perturbed. After a 24-hour CNTF treatment, not only were the rod-specific transcript levels further decreased, but the mRNAs for cone-specific *Opn1sw, Opn1mw*, and *Gnat2* were also markedly reduced. The rapid and strong CNTF impact observed on key photoreceptor transcription factors and phototransduction apparatuses is likely to cause suppression of visual function in the *Rds* mutant as observed previously^15–17^. In addition, others have reported adverse effects of CNTF on the visual cycle^37^.

A short 3-hour CNTF treatment was sufficient to cause a rapid upregulation of transcripts for key cytokine signaling components *Stat1*, *Stat3* and *Il6/gp130*, suggesting a positive regulation upon CNTF signaling. Consistent with our previous study^13^, a transient burst of the CNTF-family ligands *Lif* and *Clct1* was detected, which returned to the control *Rds* levels by 24 hours. In contrast, our showed that CNTF induces an increase of Müller glial expressed *Gfap* and *Sox9*, indicating that CNTF triggers Müller cell activation. Within 24 hours, CNTF significantly elevated transcripts of *Edn2* over the levels detected in none-treated *Rds* mutant. Edn2 is known to play an important role in photoreceptor-to-Müller glia signaling in damaged or degenerating retinas, as Edn receptor Ednrb is expressed by Müller glia^21^. It has been shown that Edn2 overexpression affect retinal vasculature by inhibiting endothelial tip cells, possibly through Müller released factors^38^. Our RNA-seq data is consistent with a central role of Müller glia in mediating CNTF effects, possibly by releasing multiple growth factors and/or cytokines. It remains to be determined whether CNTF signaling also impacts retinal vessels that can affect nutrients and oxygen supply to the neural retina.

Our gene signature pathway analyses demonstrate a prominent participation of the immune response. Compared to wild type retinas, the *Rds* mutant retina at the onset of degeneration already shows increased expression of genes involved in the innate immune system, interferon signaling, and the complement pathway. Both short- and long-term CNTF treatments further elevated the expression of genes involved in innate and adaptive immune systems, interferon, NF-kB, Jak-STAT, GPCR and MAPK signaling. Recent studies have shown a rapid and persistent activation of microglia in degenerating retinas, accompanied by significantly enhanced chemokine and cytokine gene upregulations^39–42^. Part of our RNA-seq data showing the uptick of immune system and chemokine gene expression may reflect microglia activities in the retina. However, the many changes we observed to the retinal transcriptome may also reflect CNTF-provoked responses of retinal neurons and Muller glia. By cross-validating our bulk RNA-seq data with a single-cell retinal RNA-seq dataset, we identified several retinal cell types that responded to CNTF treatment in the *Rds* retina, including rod, cone, bipolar, amacrine, and Müller glia. This result indicates that CNTF broadly impacts different retinal cell types under degeneration conditions, either directly or indirectly. It is also known that different retinal cell types express complement pathway components^43^. Our results captured changes of various complement genes, suggesting that CNTF may regulate this important system and influence their functions in the adult retina.

The mechanisms underlying long-term CNTF-mediated neuroprotection in RD are not well-understood. Interestingly, after long-term CNTF treatment, we detected altered gene signatures in cell homeostasis, ribosomal and RNA metabolism, endocytosis, ER-phagosome pathways, and transcription and translation processes. These gene signatures potentially point to cellular physiological processes influenced by CNTF to enhance neuronal viability. RNA-seq analyses may also capture novel retinal regulatory genes^27^. A recent study of retinal transcriptomes from ~500 age-related macular degeneration (AMD) cases identified ~50 AMD genes^44^. Among these, 12 genes that are involved in complement cascade and extracellular matrix organization, matching the CNTF-treated *Rds* gene signatures (*Mmp9*, *cd63*, *Dxo*, *Cfi*, *Cfh*, *Col8a1*, *Nlrc5*, *Rdh5*, *Slc16a8*, *ApoE*, *Tsc22d4*, and *Vtn*). It is intriguing that the gene signatures from mouse models can be directly associated with human RD based on genetic studies.

In summary, this study demonstrates that exogenous CNTF alters retinal transcription landscapes in a preclinical animal model of RD. Transcriptome analyses reveal dynamic and strong impacts on photoreceptors and other retinal cell types, as well as the activation of immune systems and multiple signaling pathways. This study provides a molecular simulation at the transcription level for likely responses in diseased human retinas undergoing CNTF treatment, thus shedding light towards developing new targeted therapies for blinding diseases.

## Materials and Methods

### Animals and eye injections

The *Rds/Prph2*(*P216L)* transgenic mice^9^ were maintained as hemizygotes on CD1 or C57BL/6J background. Genotyping for *Rds/Prph2*(*P216L)* were performed using tail genomic DNAs and PCR primers *5*′*CCT GGA GTT GCG CTG T*, and *5*′*GTC TTT TTC ATG AAG CAC C*. For short-term treatments, *Rds/Prph2*(*P216L)* transgenic mice with C57BL/6 background were injected intravitreally with 1 μg of recombinant rat CNTF (PeproTech) or PBS in 1 μl volume at postnatal day 25 (P25), and harvested at 3-hour or 24-hour post injections. For long-term treatment, *Rds/Prph2*(*P216L)* mice in the CD1 background were injected subretinally with lentivirus LV-CNTF or the control virus LV-IG^13^ at P25, and harvested at P35. All animal procedures followed National Institutes of Health guidelines and were approved by the Animal Research Committee at University of California Los Angeles.

### RNA extraction, amplification, and sequencing

The RNeasy RNA extraction kit was used to isolate total RNA from dissected neural retinas without the retinal pigment epithelium and other ocular tissues. On-column DNaseI digestion was performed and RNA was collected in RNase-free water, following manufacturer’s instructions (Qiagen, Hilden, Germany). The RNA quality was determined on an Agilent 2100 Bioanalyzer. All samples passed the quality filters. For short-term treatments, RNA was treated with oligo-dT beads to enrich for mRNA out of total RNA, and the libraries were preparing using Illumina TruSeq RNA Sample Preparation Kit and loaded onto a HiSeq2000 Sequencer for single-end sequencing with read lengths of 50 bases. For long-term treatment, sequencing libraries were prepared using the Illumina TruSeq Stranded Total RNA with Ribo-Zero Gold Library Prep kit. Libraries were loaded onto the HiSeq4000 Sequencer for paired-end sequencing with read length of 69 bp. The datasets are summarized in Supplementary Table S1.

### RNA sequencing data analyses

Quality of individual sequences were evaluated using the FastQC software (www.bioinformatics.babraham.ac.uk/projects/fastqc/) after adapter trimming with the cutadapt software (cutadapt.readthedocs.io/en/stable/). The reads were filtered to exclude those with more than 5% unknown bases, reads which contained more than 20% bases with quality score below 15, and reads with only adapters. After filtering, the median size was 5.65 Gb per library (range 3.66–7.26 Gb). Sequenced reads were then aligned to the Mouse reference mm10 from UCSC (genome-euro.ucsc.edu) using Hisat2 (v2.0.4)^45^. The expression levels were normalized by calculating the fragments per kilobase million reads (FPKM) values. The differential expression of transcripts was calculated using DESeq2^46^, with statistical significance *p* < 0.01. The pathway enrichment analyses were performed using GSEA^47^ and databases from the Molecular Signatures Database (MSigDB v7.0, http://www.broadinstitute.org/gsea/msigdb/).

### Validations of gene signatures using bulk RNA-seq, single-cell sequencing, and proteome data sets

To compare the gene signatures with other studies of the mouse retina, we retrieved results from previously published studies, including (1) retina RNA-seq data of two RD models^28,29^, (2) single-cell RNA-seq data of the mouse retina^31^, and (3) proteome data from the *Rd10* model^30^ (Supplementary Table S7). First, we explored diverse RD mouse models with RNA-seq transcriptomes available in the GEO database (www.ncbi.nlm.nih.gov/geo/) and retrieved studies with ≥3 biological replicates for both wild type and mutant groups (GEO accessions: GSE56473, GSE63810)^28,29^. These RNA-seq data sets were reprocessed using the Hisat2-DESeq2 pipeline described above. Second, we retrieved single-cell sequencing data of mouse retina (GEO accession: GSE81905)^31^ to evaluate which cell types are enriched in the gene signatures we detected. Third, we cross-validated the transcriptome gene signatures with *Rd10* proteome dataset at pre-, peak-, and post-degeneration time points (ProteomeXchange accession: PXD002584)^30^.

### Statistical analysis

Fisher’s exact tests with false discovery rate (FDR) correction for multiple comparisons were employed to calculate the statistical significance of the observed overlap between two gene sets. Student’s *t*-tests were performed to calculate the statistical significance between pair-wise comparisons.

## Supplementary Information

Supplementary Figure S1.

Supplementary Figure S2I.

Supplementary Legends.

Supplementary Tabe S1.

Supplementary Table S2.

Supplementary Table S3.

Supplementary Table S4.

Supplementary Table S5.

Supplementary Table S6.

Supplementary Table S7.

Supplementary References.

Supplementary Table S8.
