# Supplementary material for: Impacts of ciliary neurotrophic factor on the retinal transcriptome in a mouse model of photoreceptor degeneration
Source: Sci Rep. 2020 Apr 20;10:6593. doi: 10.1038/s41598-020-63519-1 (PMC7171121; doi:10.1038/s41598-020-63519-1)
Supplement: Supplementary file 1 — Supplementary Figure S1. [file 41598_2020_63519_MOESM1_ESM.docx]

**Supplementary Figure S1**


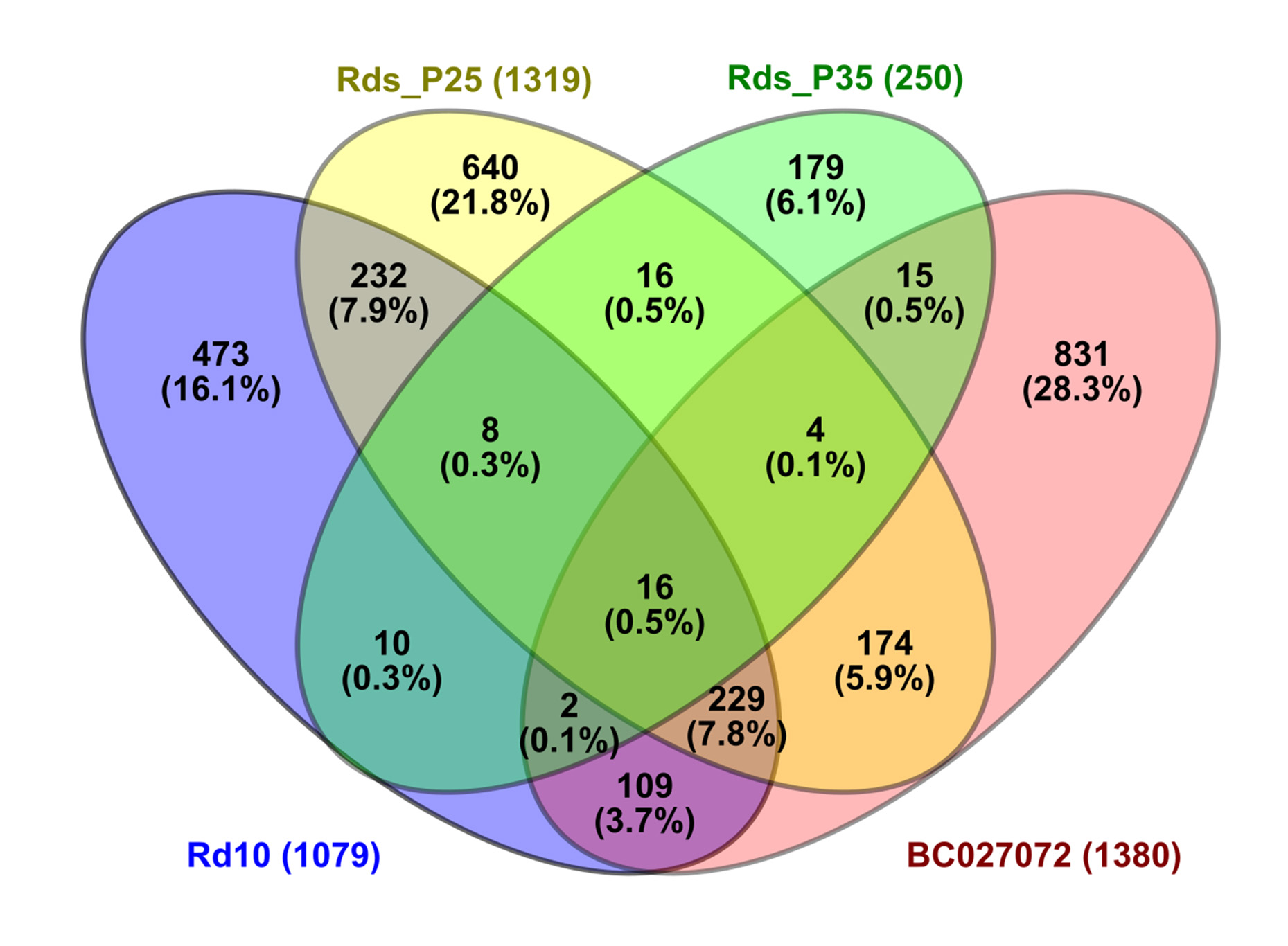


**Supplementary Figure S1. Gene signature comparisons among different retinal degeneration models.**

Venn diagram shows DEG overlaps of *Rds* retina transcriptomes at P25 and P35 with two retinal degeneration models *Rd10* and BC027072. The DEG of the three degeneration models were compared with their corresponding wild type controls. All *p* values<0.01 in Fisher's Exact Test.
