# Supplementary material for: Impacts of ciliary neurotrophic factor on the retinal transcriptome in a mouse model of photoreceptor degeneration
Source: Sci Rep. 2020 Apr 20;10:6593. doi: 10.1038/s41598-020-63519-1 (PMC7171121; doi:10.1038/s41598-020-63519-1)
Supplement: Supplementary file 2 — Supplementary Figure S2I. [file 41598_2020_63519_MOESM2_ESM.docx]

**
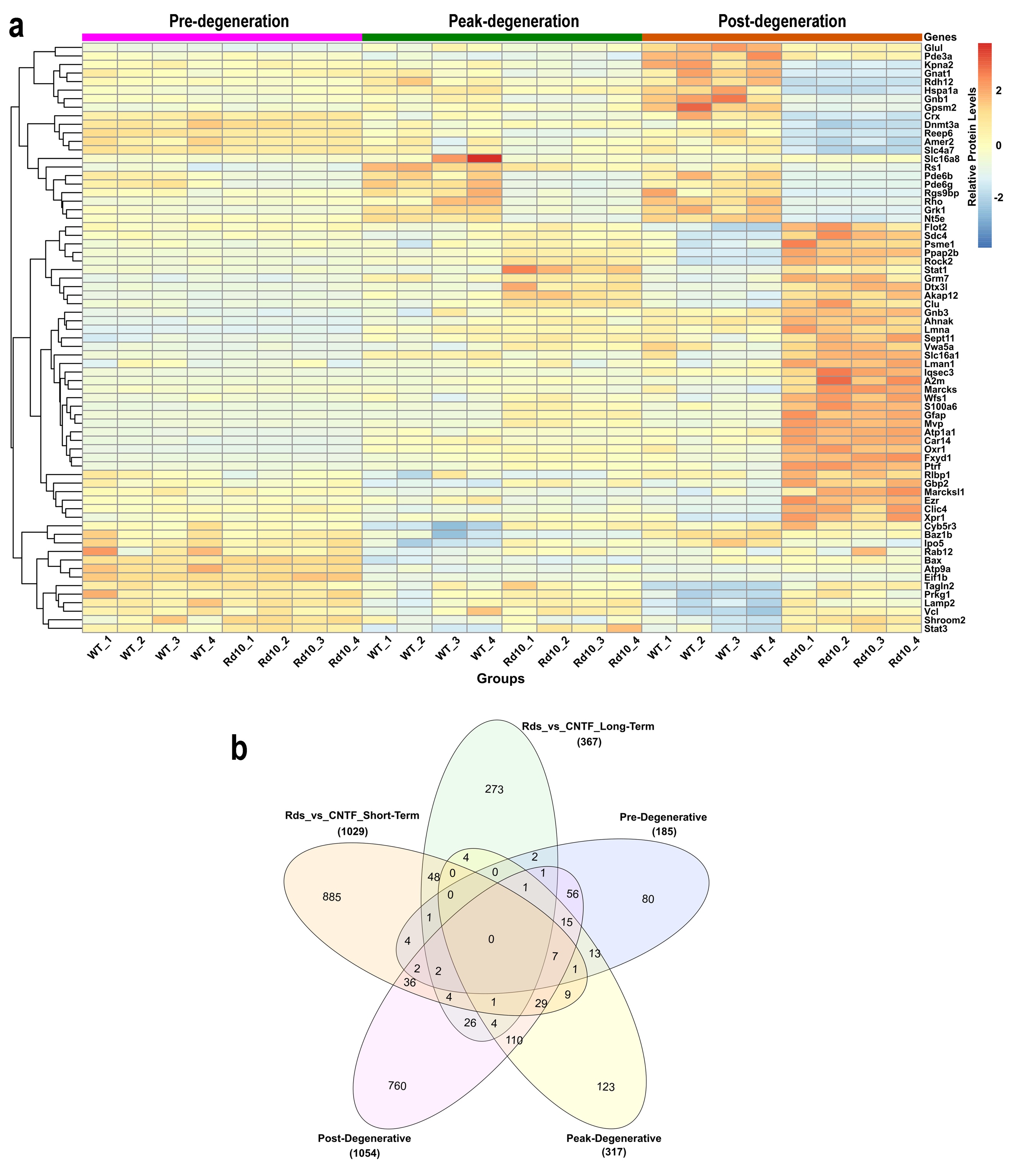
Supplementary Figure S2**

**Supplementary Figure S2. Comparison between *Rds* RNA-seq data and *Rd10* proteomic data.**

**a**) Heatmap of *Rd10* mutant retinal proteomes at pre-, peak-, and post-degeneration stages is shown. The selected proteins listed in the heatmap match the significant RNA-seq DEG of *Rds* retinas treated with CNTF (short- and long-term treatments combined). **b**) Venn diagram shows the overlapping of significant RNA-seq DEGs from short-and long-term CNTF treated *Rds* retinas with the *Rd10* proteomes. All *p* values<0.01 in Fisher's Exact Test.
