# Supplementary material for: Impacts of ciliary neurotrophic factor on the retinal transcriptome in a mouse model of photoreceptor degeneration
Source: Sci Rep. 2020 Apr 20;10:6593. doi: 10.1038/s41598-020-63519-1 (PMC7171121; doi:10.1038/s41598-020-63519-1)
Supplement: Supplementary file 3 — Supplementary Legends. [file 41598_2020_63519_MOESM3_ESM.docx]

**SUPPLEMENTARY INFORMATION**

**Impacts of ciliary neurotrophic factor on the retinal transcriptome in a mouse model**

**of photoreceptor degeneration**

Yanjie Wang^1^, Kun-Do Rhee^1^, Matteo Pellegrini^2^, Xian-Jie Yang^1*^

^1^Department of Ophthalmology and Stein Eye Institute, ^2^Department of Molecular, Cell and Developmental Biology, University of California, Los Angeles, CA, USA

**Supplementary Table S1**. Summary of retinal samples and high throughput sequencing

**Supplementary Table S2**. Gene signatures in short-term CNTF treatments

**Supplementary Table S3.** Pathway enrichment of gene signatures detected in short-term CNTF treatments

**Supplementary Table S4.** Expression levels of selected retinal genes in short-term CNTF treated samples

**Supplementary Table S5.** Gene signatures in the long-term CNTF treated Rds retinas

**Supplementary Table S6.** Pathway enrichment of gene signatures detected in the long-term CNTF treated Rds retinas

**Supplementary Table S7.** Summary of datasets used for validation of gene signatures

**Supplementary Table S8.** Gene signatures of mouse retina degeneration models

**Supplementary Figure S1**. Gene signature comparisons among different retinal degeneration models

**Supplementary Figure S2**. Comparison between *Rds* RNA-seq data and *Rd10* proteomic data
