# Supplementary material for: Impacts of ciliary neurotrophic factor on the retinal transcriptome in a mouse model of photoreceptor degeneration
Source: Sci Rep. 2020 Apr 20;10:6593. doi: 10.1038/s41598-020-63519-1 (PMC7171121; doi:10.1038/s41598-020-63519-1)
Supplement: Supplementary file 4 — Supplementary Tabe S1. [file 41598_2020_63519_MOESM4_ESM.docx]

**Supplementary Table S1**

**Summary of Retinal Samples and High Throughput Sequencing**

| **Treatments** | **Groups** | **Sample Size** | **Instrument** | **Library Type** | **Read Lengths** | **Read Counts** |
| --- | --- | --- | --- | --- | --- | --- |
| Long-Term  P25-P35 | *Rds(P216L)*  LV-hCNTF | 3 | HiSeq4000 | All RNA except rRNA | 69 bp paired-end | 9.37E8 |
|  | *Rds(P216L)*  LV-IG | 3 | HiSeq4000 | All RNA except rRNA | 69 bp paired-end | 9.71E8 |
|  | Wild Type | 3 | HiSeq4000 | All RNA except rRNA | 69 bp paired-end | 7.53E8 |
| Short-Term  P25 | *Rds(P216L)*  CNTF, 3 h | 2 | HiSeq2000 | Poly-A selection | 50 bp  single-end | 2.24E7 |
|  | *Rds(P216L)*  PBS, 3 h | 2 | HiSeq2000 | Poly-A selection | 50 bp  single-end | 2.12E7 |
|  | *Rds(P216L)*  CNTF, 24 h | 2 | HiSeq2000 | Poly-A selection | 50 bp  single-end | 2.27E7 |
|  | *Rds(P216L)*  PBS, 24 h | 2 | HiSeq2000 | Poly-A selection | 50 bp  single-end | 2.52E7 |
|  | *Rds(P216L)* | 2 | HiSeq2000 | Poly-A selection | 50 bp  single-end | 2.49E7 |
|  | Wild Type | 2 | HiSeq2000 | Poly-A selection | 50 bp  single-end | 2.28E7 |
