# Supplementary material for: Impacts of ciliary neurotrophic factor on the retinal transcriptome in a mouse model of photoreceptor degeneration
Source: Sci Rep. 2020 Apr 20;10:6593. doi: 10.1038/s41598-020-63519-1 (PMC7171121; doi:10.1038/s41598-020-63519-1)
Supplement: Supplementary file 10 — Supplementary Table S7. [file 41598_2020_63519_MOESM10_ESM.docx]

**Supplementary Table S7**

**Summary of Datasets Used for Validation of Gene Signatures**

| **Data Accessions** | **Types** | **Descriptions** | **Sample Size** | **Protocols** |
| --- | --- | --- | --- | --- |
| GSE56473 | RNA-seq | *Rd10* transcriptome | 3 *Rd10* vs. 3 WT | Hisat2 - Stringtie - DESeq2 |
| GSE63810 | RNA-seq | BC027072 -/- transcriptome | 3 KO vs. 3 WT | Hisat2 - Stringtie - DESeq2 |
| GSE81905 | Single-cell sequencing | Drop-seq of retinal cells | ~45,000 cells | STAR - t-SNE |
| PXD002584 | Proteome | *Rd10* retina proteome | 4 *Rd10* vs. 4 WT at pre-, peak-, and post-degenerative time points | Student’s t-test |
