# Supplementary material for: Impacts of ciliary neurotrophic factor on the retinal transcriptome in a mouse model of photoreceptor degeneration
Source: Sci Rep. 2020 Apr 20;10:6593. doi: 10.1038/s41598-020-63519-1 (PMC7171121; doi:10.1038/s41598-020-63519-1)
Supplement: Supplementary file 11 — Supplementary References. [file 41598_2020_63519_MOESM11_ESM.docx]

**References**

1 Wen, R., Tao, W., Li, Y. & Sieving, P. A. CNTF and retina. *Progress in retinal and eye research* **31**, 136-151, doi:10.1016/j.preteyeres.2011.11.005 (2012).

2 Sieving, P. A. *et al.* Ciliary neurotrophic factor (CNTF) for human retinal degeneration: phase I trial of CNTF delivered by encapsulated cell intraocular implants. *Proceedings of the National Academy of Sciences of the United States of America* **103**, 3896-3901, doi:10.1073/pnas.0600236103 (2006).

3 Zhang, K. *et al.* Ciliary neurotrophic factor delivered by encapsulated cell intraocular implants for treatment of geographic atrophy in age-related macular degeneration. *Proceedings of the National Academy of Sciences of the United States of America* **108**, 6241-6245, doi:10.1073/pnas.1018987108 (2011).

4 Birch, D. G. *et al.* Randomized trial of ciliary neurotrophic factor delivered by encapsulated cell intraocular implants for retinitis pigmentosa. *American journal of ophthalmology* **156**, 283-292 e281, doi:10.1016/j.ajo.2013.03.021 (2013).

5 Zein, W. M. *et al.* CNGB3-achromatopsia clinical trial with CNTF: diminished rod pathway responses with no evidence of improvement in cone function. *Investigative ophthalmology & visual science* **55**, 6301-6308, doi:10.1167/iovs.14-14860 (2014).

6 Chew, E. Y. *et al.* Effect of Ciliary Neurotrophic Factor on Retinal Neurodegeneration in Patients with Macular Telangiectasia Type 2: A Randomized Clinical Trial. *Ophthalmology* **126**, 540-549, doi:10.1016/j.ophtha.2018.09.041 (2019).

7 Scerri, T. S. *et al.* Genome-wide analyses identify common variants associated with macular telangiectasia type 2. *Nat Genet* **49**, 559-567, doi:10.1038/ng.3799 (2017).

8 Chang, E. E. & Goldberg, J. L. Glaucoma 2.0: neuroprotection, neuroregeneration, neuroenhancement. *Ophthalmology* **119**, 979-986, doi:10.1016/j.ophtha.2011.11.003 (2012).

9 Kedzierski, W., Lloyd, M., Birch, D. G., Bok, D. & Travis, G. H. Generation and analysis of transgenic mice expressing P216L-substituted rds/peripherin in rod photoreceptors. *Invest Ophthalmol Vis Sci* **38**, 498-509 (1997).

10 Boon, C. J. *et al.* The spectrum of retinal dystrophies caused by mutations in the peripherin/RDS gene. *Prog Retin Eye Res* **27**, 213-235, doi:10.1016/j.preteyeres.2008.01.002 (2008).

11 Rhee, K. D., Goureau, O., Chen, S. & Yang, X. J. Cytokine-induced activation of signal transducer and activator of transcription in photoreceptor precursors regulates rod differentiation in the developing mouse retina. *The Journal of neuroscience : the official journal of the Society for Neuroscience* **24**, 9779-9788, doi:10.1523/JNEUROSCI.1785-04.2004 (2004).

12 Ip, N. Y. The neurotrophins and neuropoietic cytokines: two families of growth factors acting on neural and hematopoietic cells. *Ann N Y Acad Sci* **840**, 97-106 (1998).

13 Rhee, K. D. *et al.* CNTF-mediated protection of photoreceptors requires initial activation of the cytokine receptor gp130 in Muller glial cells. *Proceedings of the National Academy of Sciences of the United States of America* **110**, E4520-4529, doi:10.1073/pnas.1303604110 (2013).

14 Schlichtenbrede, F. C. *et al.* Intraocular gene delivery of ciliary neurotrophic factor results in significant loss of retinal function in normal mice and in the Prph2Rd2/Rd2 model of retinal degeneration. *Gene therapy* **10**, 523-527, doi:10.1038/sj.gt. 3301929 [pii] (2003).

15 Bok, D. *et al.* Effects of adeno-associated virus-vectored ciliary neurotrophic factor on retinal structure and function in mice with a P216L rds/peripherin mutation. *Experimental eye research* **74**, 719-735, doi:S0014483502911760 [pii] (2002).

16 Bush, R. A. *et al.* Encapsulated cell-based intraocular delivery of ciliary neurotrophic factor in normal rabbit: dose-dependent effects on ERG and retinal histology. *Invest Ophthalmol Vis Sci* **45**, 2420-2430 (2004).

17 Rhee, K. D. *et al.* Molecular and cellular alterations induced by sustained expression of ciliary neurotrophic factor in a mouse model of retinitis pigmentosa. *Investigative ophthalmology & visual science* **48**, 1389-1400, doi:10.1167/iovs.06-0677 (2007).

18 Rao, J. *et al.* ATF3-mediated NRF2/HO-1 signaling regulates TLR4 innate immune responses in mouse liver ischemia/reperfusion injury. *Am J Transplant* **15**, 76-87, doi:10.1111/ajt.12954 (2015).

19 Song, L. *et al.* BCL3 Reduces the Sterile Inflammatory Response in Pancreatic and Biliary Tissues. *Gastroenterology* **150**, 499-512 e420, doi:10.1053/j.gastro.2015.10.017 (2016).

20 Awan, B. *et al.* FGF2 Induces Migration of Human Bone Marrow Stromal Cells by Increasing Core Fucosylations on N-Glycans of Integrins. *Stem Cell Reports* **11**, 325-333, doi:10.1016/j.stemcr.2018.06.007 (2018).

21 Rattner, A. & Nathans, J. The genomic response to retinal disease and injury: evidence for endothelin signaling from photoreceptors to glia. *J Neurosci* **25**, 4540-4549, doi:10.1523/JNEUROSCI.0492-05.2005 (2005).

22 Furukawa, T., Morrow, E. M. & Cepko, C. L. Crx, a novel otx-like homeobox gene, shows photoreceptor-specific expression and regulates photoreceptor differentiation. *Cell* **91**, 531-541, doi:10.1016/s0092-8674(00)80439-0 (1997).

23 Rehemtulla, A. *et al.* The basic motif-leucine zipper transcription factor Nrl can positively regulate rhodopsin gene expression. *Proceedings of the National Academy of Sciences of the United States of America* **93**, 191-195, doi:10.1073/pnas.93.1.191 (1996).

24 Mears, A. J. *et al.* Nrl is required for rod photoreceptor development. *Nat Genet* **29**, 447-452, doi:10.1038/ng774 (2001).

25 Chen, S. *et al.* Crx, a novel Otx-like paired-homeodomain protein, binds to and transactivates photoreceptor cell-specific genes. *Neuron* **19**, 1017-1030, doi:10.1016/s0896-6273(00)80394-3 (1997).

26 Wright, A. F. *et al.* Mutation analysis of NR2E3 and NRL genes in Enhanced S Cone Syndrome. *Hum Mutat* **24**, 439, doi:10.1002/humu.9285 (2004).

27 Albrecht, N. E. *et al.* Rapid and Integrative Discovery of Retina Regulatory Molecules. *Cell Rep* **24**, 2506-2519, doi:10.1016/j.celrep.2018.07.090 (2018).

28 Kevany, B. M., Zhang, N., Jastrzebska, B. & Palczewski, K. Animals deficient in C2Orf71, an autosomal recessive retinitis pigmentosa-associated locus, develop severe early-onset retinal degeneration. *Hum Mol Genet* **24**, 2627-2640, doi:10.1093/hmg/ddv025 (2015).

29 Uren, P. J., Lee, J. T., Doroudchi, M. M., Smith, A. D. & Horsager, A. A profile of transcriptomic changes in the rd10 mouse model of retinitis pigmentosa. *Mol Vis* **20**, 1612-1628 (2014).

30 Ly, A. *et al.* Proteomic Profiling Suggests Central Role Of STAT Signaling during Retinal Degeneration in the rd10 Mouse Model. *J Proteome Res* **15**, 1350-1359, doi:10.1021/acs.jproteome.6b00111 (2016).

31 Shekhar, K. *et al.* Comprehensive Classification of Retinal Bipolar Neurons by Single-Cell Transcriptomics. *Cell* **166**, 1308-1323 e1330, doi:10.1016/j.cell.2016.07.054 (2016).

32 Wert, K. J., Lin, J. H. & Tsang, S. H. General pathophysiology in retinal degeneration. *Dev Ophthalmol* **53**, 33-43, doi:10.1159/000357294 (2014).

33 Chang, B. Mouse models for studies of retinal degeneration and diseases. *Methods Mol Biol* **935**, 27-39, doi:10.1007/978-1-62703-080-9_2 (2013).

34 Chang, B. *et al.* Retinal degeneration mutants in the mouse. *Vision Res* **42**, 517-525 (2002).

35 Rashid, K., Dannhausen, K. & Langmann, T. Testing for Known Retinal Degeneration Mutants in Mouse Strains. *Methods Mol Biol* **1834**, 45-58, doi:10.1007/978-1-4939-8669-9_3 (2019).

36 Farkas, M. H., Au, E. D., Sousa, M. E. & Pierce, E. A. RNA-Seq: Improving Our Understanding of Retinal Biology and Disease. *Cold Spring Harb Perspect Med* **5**, a017152, doi:10.1101/cshperspect.a017152 (2015).

37 Li, S. *et al.* Ciliary neurotrophic factor (CNTF) protects retinal cone and rod photoreceptors by suppressing excessive formation of the visual pigments. *J Biol Chem* **293**, 15256-15268, doi:10.1074/jbc.RA118.004008 (2018).

38 Rattner, A., Yu, H., Williams, J., Smallwood, P. M. & Nathans, J. Endothelin-2 signaling in the neural retina promotes the endothelial tip cell state and inhibits angiogenesis. *Proc Natl Acad Sci U S A* **110**, E3830-3839, doi:10.1073/pnas.1315509110 (2013).

39 Anderson, S. R. & Vetter, M. L. Developmental roles of microglia: A window into mechanisms of disease. *Dev Dyn* **248**, 98-117, doi:10.1002/dvdy.1 (2019).

40 Wang, X. *et al.* Requirement for Microglia for the Maintenance of Synaptic Function and Integrity in the Mature Retina. *J Neurosci* **36**, 2827-2842, doi:10.1523/JNEUROSCI.3575-15.2016 (2016).

41 Silverman, S. M. & Wong, W. T. Microglia in the Retina: Roles in Development, Maturity, and Disease. *Annu Rev Vis Sci* **4**, 45-77, doi:10.1146/annurev-vision-091517-034425 (2018).

42 Zhang, Y. *et al.* Repopulating retinal microglia restore endogenous organization and function under CX3CL1-CX3CR1 regulation. *Sci Adv* **4**, eaap8492, doi:10.1126/sciadv.aap8492 (2018).

43 Pauly, D. *et al.* Cell-Type-Specific Complement Expression in the Healthy and Diseased Retina. *Cell Rep* **29**, 2835-2848 e2834, doi:10.1016/j.celrep.2019.10.084 (2019).

44 Ratnapriya, R. *et al.* Retinal transcriptome and eQTL analyses identify genes associated with age-related macular degeneration. *Nat Genet* **51**, 606-610, doi:10.1038/s41588-019-0351-9 (2019).

45 Pertea, M., Kim, D., Pertea, G. M., Leek, J. T. & Salzberg, S. L. Transcript-level expression analysis of RNA-seq experiments with HISAT, StringTie and Ballgown. *Nat Protoc* **11**, 1650-1667, doi:10.1038/nprot.2016.095 (2016).

46 Love, M. I., Huber, W. & Anders, S. Moderated estimation of fold change and dispersion for RNA-seq data with DESeq2. *Genome Biol* **15**, 550, doi:10.1186/s13059-014-0550-8 (2014).

47 Subramanian, A. *et al.* Gene set enrichment analysis: a knowledge-based approach for interpreting genome-wide expression profiles. *Proc Natl Acad Sci U S A* **102**, 15545-15550, doi:10.1073/pnas.0506580102 (2005).
